# Point-of-Care Portable 3D-Printed Multispectral Sensor for Real-Time Enzyme Activity Monitoring in Healthcare Applications

**DOI:** 10.3390/bios13010120

**Published:** 2023-01-10

**Authors:** Antony Jesuraj, Umer Hassan

**Affiliations:** 1 Department of Electrical and Computer Engineering, School of Engineering, Rutgers, The State University of New Jersey, New Brunswick, NJ 08854, USA; 2 Global Health Institute, Rutgers, The State University of New Jersey, New Brunswick, NJ 08901, USA

**Keywords:** absorbance spectroscopy, enzyme kinetics, enzyme inhibition, multispectral sensing

## Abstract

Absorbance spectroscopy finds many biomedical and physical applications ranging from studying the atomic and molecular details of the analyte to determination of unknown biological species and their concentration or activity in the samples. Commercially available laboratory-based spectrometers are usually bulky and require high power and laborious manual processing, making them unsuitable to be deployed in portable and space-constrained environments, thereby further limiting their utility for real-time on-site monitoring. To address these challenges, here we developed a portable 3D-printed multispectral spectrophotometer based on absorbance spectroscopy for real-time monitoring of enzyme molecular activity. Monitoring enzyme (such as tyrosinase) activity is critical, as it quantifies its reaction rate, which is dependent on many factors such as the enzyme and substrate concentrations, temperature, pH, and other regulators such as inhibitors and effectors. Tyrosinase is a critical enzyme responsible for melanin synthesis in living beings and exhibits enzymatic browning in fruits and vegetables. It finds various commercial applications in the fields of healthcare (skin pigmentation, wound healing, etc.), forensics, and food processing. Here, tyrosinase activity was monitored using a 3D-printed spectral sensor at different rates and compared against measurements obtained from laboratory instruments. The enzyme activity was also studied using kojic acid (i.e., a commonly employed commercial tyrosinase inhibitor) while varying its molar and volume concentrations to control the reaction rate at discrete activity levels. For tyrosinase activity monitoring, the fabricated device has shown significant correlation (R^2^ = 0.9999) compared to measurements from the standard table-top spectrophotometer. We also provide a performance comparison between the 3D-printed and the laboratory spectrophotometer instruments by studying tyrosinase enzyme activity with and without the influence of an inhibitor. Such a device can be translated into various absorbance spectroscopy-based point-of-care biomedical and healthcare applications.

## 1. Introduction

Absorbance spectroscopy has applications ranging from studying the atomic and molecular details of the analyte to determining an unknown substance(s) and its concentration in the analyte. The absorption spectrum of the analyte will depend on the excitation photon energy and the electronic energy levels within the analyte. The range of excitation wavelength used depends on the type of sample under study and the allowed electron transition states. Absorption spectroscopy typically requires the incident EM wave to interact with the sample under study and, further, requires the energy of the incident wave to be equal to the energy bandgap between the ground state and the excited state of the molecule. In this paper, transmission mode measurement is utilized to perform absorbance spectroscopy on enzymes to study their kinetics in real time by implementing Beer–Lambert’s law. Enzymes are responsible for catalyzing various biochemical reactions depending on their molecular structure, resulting in a new biochemical product. Depending on the optical properties of the resulting product, different measurement techniques can be employed to perform their monitoring. Enzymes are broadly classified into seven categories. They are: oxidoreductase (involving electron transfer during the reaction), hydrolase (this breaks chemical bonds using water molecule), isomerase (involves structural rearrangement of the molecule during the reaction), ligase (establishes a new chemical bond while catalyzing the reaction of joining two large molecules), transferase (transfers a specific functional atom during the reaction), lyase (breaks a chemical bond by hydrolysis or oxidation during the reaction), and translocase (catalyzes the reaction by assisting the movement of the molecules) [1]. Among these, tyrosinase (of the oxidoreductase type) plays a vital role in the formation of melanin in humans and animals [2,3]. Further, considering its huge utility in a range of applications, there are several cases where inhibiting enzyme activity is of critical importance too, e.g., in commercial skin care products [4]. Tyrosinase is responsible for the formation of melanin by oxidation of an amino acid such as L-Dopa, also referred to as a substrate. The formation of melanin catalyzed by tyrosinase happens in two stages: first, hydroxylation of monophenols to form O–diphenol, and second, oxidation of O–diphenol to O–quinones. The dopachrome formed has a maximum absorbance at 475 nm and, with an additional series of chemical reactions, gets converted into melanin. Two types of assays were performed to understand enzyme kinetics using a spectrophotometer for activity monitoring while using L-Dopa as a substrate [5]. For melanin synthesis, tyrosinase oxidizes amino acids to produce dopaquinone, exhibiting a kinetic reaction. These enzymes are essentially oxidoreductases that catalyze the biochemical reaction, converting phenols to melanin in a series of reaction involving hydroxylation and oxidation. This process is also termed melanogenesis. The produced dopaquinone or dopachrome is optically sensitive at 475 nm, and monitoring its absorbance data at specified time intervals can indicate the rate of synthesis, indicating the enzyme kinetics for a known type of substrate. Fan et al. studied tyrosinase assay using different techniques and found the spectrophotometric method to have high performance and accuracy, thereby highlighting the utility of fluorescent substrates towards fluorescent imaging [6]. Different chemical reactions are often monitored to understand reaction kinetics, the property of the biproducts formed, and the consumption rate of the substrate responsible for the reaction. Braunschweig et al. employed chemical force microscopy to monitor tyrosinase enzyme activity using AFM as an analytical tool [7]. While many techniques were evaluated in the past, optical spectroscopy is one of the most employed methods to monitor enzyme activity. Wang et al. described a portable pigment measurement system using optical fibers interfaced with the measurement cavity [8]. This technique utilized an external commercial spectrophotometer that was interfaced with optical fibers to the measurement cavity. Kittipanyangam et al. developed a color light sensor to measure light absorbance based on the response from RGB sensitive detectors with the limitation of narrow wavelength spectral measurement [9]. Similarly, Murdock et al. developed a method to make stable measurements by utilizing a background measurement solution to minimize errors while performing optical spectroscopy [10]. In another study, a biofilm using *E. coli* was prepared and studied using optical absorbance to monitor the growth of the cells. However, this required external interfaces to measure optical absorbance [11]. Recently, our group had also developed a portable 3D-printed spectrophotometer based on the absorbance spectroscopy principle for monitoring rifampicin concentration in buffers and synthetic urine for therapeutic drug monitoring [12].

Tyrosinase’s utility in commercial applications requires its extraction from several sources [13]. The extraction process and its utility in subsequent applications requires tyrosinase activity monitoring for quality assessment of the processes involved. The broad commercial and healthcare applications further highlight the need for an accurate spectrophotometer. Different research groups in the past have developed spectrophotometers with varying levels of automation. Nguyen et.al studied tyrosinase inhibitory activity using an extract of the wood of *Artocarpus heterophyllous*. This study was performed using laboratory equipment at 475 nm [14]. Similarly, Wang et al. performed a spectroscopic study to monitor the inhibitory effect of morin on tyrosinase by performing absorbance measurements at 475 nm every 20 s [15]. Kim et al. performed a fluorescence quenching study on tyrosinase under the influence of inhibitors to measure enzyme activity using a laboratory spectrometer [16]. Recently, microfluidic devices have also been developed for spectroscopy measurements. For example, Krishnaswamy et al. developed a microfluidic integrated absorbance measurement setup with an integrated lab-on-a-chip sensor to analyze fluids when passed through the channel [17]. Usuba et al. developed a similar device using polydimethylsiloxane (PDMS) integrated with a micro lens and optical fibers and captured antibodies in the measurement area [18]. Lu et al. performed absorbance measurements to sense gas presence and concentration using a photodetector [19]. Pavithra et al. demonstrated a tyrosinase based detector for dopamine using fluorescence spectroscopy with an excitation wavelength of 230 nm and measured the response at 330 nm [20]. Luo et al. used a laboratory spectrometer to detect tyrosinase-based biomarkers using carbon dots while measuring the response at 320 nm [21].

Some of the commercially available spectrophotometers and their form factors are J-815, used in UV-Vis absorbance mode (1115 mm × 576 mm × 410 mm), Shimatzu UV-2450 (1117 mm × 711 mm × 520 mm), UH4150 (680 mm × 470 mm × 300 mm), UV1600 (460 mm × 360 mm × 225 mm), Perkin Elmer Lambda 35 (650 mm × 56 mm × 233 mm), and HR2000+ (148.6 mm × 104.8 mm × 45.1 mm). These are table-top bench instruments and weigh over 15 kg, except for the HR2000+, which is a commercially available portable spectrometer weighing about 500 mg and uses an optical fiber interface to the measurement cavity. In contrast, the 3D-printed device discussed in this paper has overall dimensions of 132 mm × 62 mm × 39 mm, including the measurement cavity used for monitoring tyrosinase enzyme activity in real time. Further, our device has the flexibility to configure the excitation and the measurement features and therefore can be used for fluorescence measurement.

Enzyme activity is also studied under the influence of an inhibitor and is compared with the performance of the laboratory instrument spectrophotometer for the same reaction–mix samples. The control instrument used throughout the measurements is UV-1600 to generate the absorbance values in kinetics mode. The portable device presented in this paper can perform both photometry and kinetics measurement at a range of excitation wavelengths, which will expand its application to various types of future studies.

## 2. Materials and Methods

### 2.1. Design of the Portable Spectrophotometer

The 3D-printed multispectral spectrophotometer is an integrated system comprising a constant, current-driven excitation source, i.e., a light emitting diode (LED, manufacturer part number: C566D-BFE-CU34Q4S2) and a digital multispectral detector (manufacturer part number: AS7341-DLGT). The excitation LED had its maximum emission spectrum at 475 nm. An 11-channel multispectral sensor covering approximately the visible spectrum (350 nm to 1000 nm) with integrated optical filters of Gaussian characteristics is used for discrete spectral range measurement. Each of these channels corresponds to an individual photodetector in combination with its optical filter packaged within the device aperture. This device was powered using universal serial bus (USB) at 5 V and consists of a power management integrated circuit (PMIC) to step down the voltage as per the subsystem requirement. The integrated electronic system was packaged within a 3D-printed enclosure that accounts for the optical design considering the aperture of the excitation beam used for absorbance spectroscopy. The measurement cavity was designed to hold microcuvettes that are capable of holding a range of sample volumes. However, for this study, a 1.5 mL cuvette with 10 mm path length is used throughout the enzyme activity measurement.

**Figure 1:**
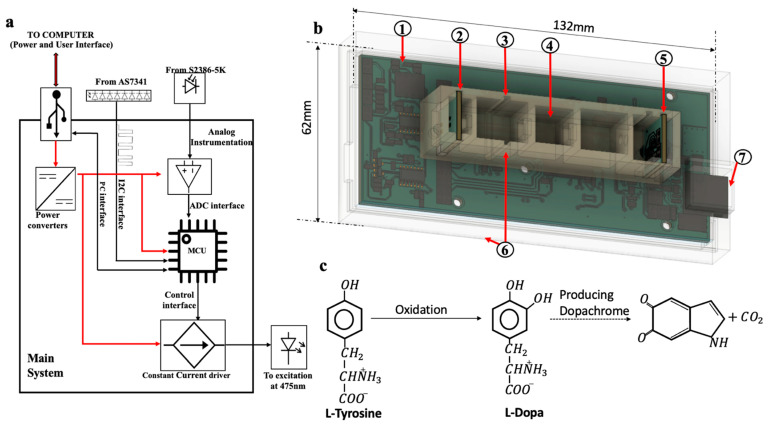
The overall system monitoring enzyme kinetics and the reaction theory is shown. (**a**) The overall system block diagram is shown along with its respective interfaces. (**b**) The 3D design isometric view of the spectrophotometer is shown with the packaging profile indicating different major subsections. The representations are as follows: (1) master PCB, (2) excitation source, (3) slot for optical filter, (4) measurement cavity to place the sample, (5) detector daughter PCB, (6) outer enclosure, (7) USB interface. (**c**) The chemical reaction of the enzyme.

The excitation source and the detector were assembled and mounted on a custom fabricated printed circuit board (PCB) to ensure the light beam was focused within the measurement cavity. These daughter boards were interfaced with the master board to achieve overall system functionality. The master board was designed to have a user interface with a personal computer (PC), power converters, microcontroller for processing, a secondary microcontroller with USB controller, constant current driver for LED excitation, ADC instrumentation, and additional ports. This is indicated in Figure 1a where the user interface to the PC consists of a common interface for both power and data. Fusion 360 CAD software by Autodesk (San Francisco, CA, USA) was used to design the enclosure for the system packaging the master board and the respective daughter boards. Eagle software by Autodesk (San Francisco, CA, USA) was used to design the electronic circuit boards. The complete design is shown in Figure 1b in isometric view, indicating the major parts of the 3D-printed spectrophotometer. There are multiple slots presented across the measurement cavity to test different configurations using optical filters and lenses. The enclosure was 3D-printed using the filament deposition modeling (FDM) technique, and polylactic acid (PLA) used as the filament during fabrication. The gain and the integration time of the detector are the most important configurations for adjusting the sensitivity of the detector. In this experiment, the integration time was set to 2.78 μs and gain to 256× for precise and maximum sensitivity using the software configurations. These measurements were then logged in the computer via USB. Tyrosinase converts the substrate (L-Dopa) into dopachrome (O–Di- quinone) by the hydroxylation and oxidation processes. The chemical equation corresponding to this is shown in Figure 1c. The produced red-colored dopachrome has maximum absorbance at 475 nm. This is captured by the one of the channels from the detector whose center wavelength is at 480 nm and whose full width half maximum is about 36 nm.

### 2.2. Absorbance and Unknown Concentration Calculation

The unknown concentration of the sample can be measured from the absorbance value using the spectrophotometer, using the calculations performed based on the principle of Beer–Lambert’s law. This law states that the absorbance of the solution is linearly related to its concentration. The equation is as follows:

(1) A=ϵ×C × P

where A is the absorbance, ϵ is the molar absorptivity or molar absorption coefficient, C is the molar concentration of the solute in the solution, and P is the optical path length of the sample. The 3D-printed spectrophotometer can self-calibrate using the dark measurement performed before placing the sample inside the measurement cavity for the optical absorbance measurement in order to monitor the enzyme activity. ADC counts are generated by the detector corresponding to the absorbance of the sample and are obtained by the microcontroller by reading the memory registers. These measured values are then converted to absorbance with respect to the dark measurement corresponding to the difference between the dark measurement and the sample measurement. This may also correspond to the intensity change due to the light absorbed by the sample during enzyme activity. The absorbance can be measured based on the intensity that the detector measures as follows:

(2) $$A=\log_{10}\left(\frac{I_{0}}{I}\right)$$

where A is the absorbance, $I_{0}$ is the intensity of the incident light, and I is the intensity of the transmitted light through the sample. $I_{0}$ is used as the reference measurement or blank measurement value for the absorbance calculation.

### 2.3. Enzyme Activity Measurement from Absorbance

The protocol used for the absorbance measurements for enzyme activity determination relied on taking measurements until the absorbance saturation point was reached. First, reaction–mix solution was transferred into both the 3D-printed spectrophotometer and the control laboratory spectrophotometer simultaneously for comparative study. Enzyme activity corresponds to the amount of the enzyme that catalyzes the substrate to transform 1 μmole into product per minute under the same specified physical conditions during the reaction. The enzyme activity is calculated using the following equation:

(3) $$Activity=\left(\frac{\Delta A}{\Delta t}\right)*\left(\frac{1}{\epsilon P}*10^{6}*V\right)$$

where ΔA is the change in the absorbance value over unit time Δt (minutes or seconds), ϵ is the molar absorptivity or molar absorption coefficient, P is the optical path length (cm), and V is the final volume of the mix solution (L).

In Equation (3), many parameters are constant and predefined. These definitions are at specific conditions of pH, temperature, and substrate concentration during enzyme activity monitoring. The change in absorbance value is calculated over the region of the kinetics where there is maximum change observed and before the enzyme saturates during the reaction. In this study, the absorbance readings measured in transmission mode are dependent on various parameters that include the path length of the sample, intensity of the incident light, concentration of the enzyme, and the molar absorption coefficient. The unit for activity is μmol min^−1^. The molar absorption coefficient for dopachrome is 3600 mol^−1^ L cm^−1^. V is 3 mL for the experiments performed. Hence, Equation (3) can be rewritten as follows:

(4) $$Activity=\left(\frac{\Delta A}{\Delta t}\right)*0.833$$

where 0.833 is calculated from the constants mentioned above, which is also known as tyrosinase activity factor.

### 2.4. Tyrosinase Enzyme Kinetics

Tyrosinase exhibits kinetics reactions when L-Dopa produces dopachrome. This reaction is dependent on various parameters such as the enzyme and substrate concentration, temperature, pH, and regulators. Enzyme kinetics is measured at different enzyme volume concentrations. Enzyme assay used following chemical reagents in the experiments: 1× PBS (Gibco PBS pH 7.4), tyrosinase (T3824-50KU from Millipore Sigma, Burlington, MA, USA), and L- 3,4-dihydroxyphenylalanine (D9628-25G from Millipore Sigma), also known as L-Dopa. The tyrosinase stock solution of concentration 5 units/μL in 1× PBS was prepared. This stock solution was refrigerated after it becomes completely soluble in PBS. The stock solution for L-Dopa was prepared with a concentration of 2 mg/mL in 1× PBS. Enzyme activity was measured at different volume concentrations of the tyrosinase solution. A 3 mL reaction mix solution was prepared for each measurement run with 60 μL, 40 μL, 20 μL, 10 μL, and 5 μL of tyrosinase solution with a constant 1 mL L-Dopa solution throughout the assay. Volumes used for 1× PBS were 1.940 mL, 1.960 mL, 1.980 mL, 1.990 mL, and 1.995 mL, respectively, within the reaction mix solutions. For each of these samples prepared for monitoring the kinetics measurement, absorbance spectroscopy was performed at 475 nm excitation, and the response was measured at 470–490 nm because of the maximum absorbance at 475 nm of the dopachrome product produced during the reaction. During the enzyme kinetics study, a second sample was measured with same reagent concentrations and conditions, and both samples were individually monitored by both the 3D-printed spectrophotometer and the control laboratory instrument. All measurements were done in triplicate.

**Figure 2:**
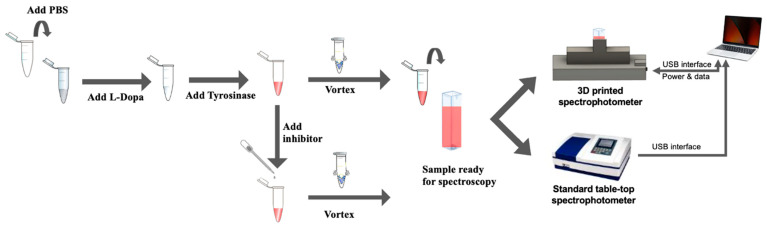
The assay protocol to monitor enzyme kinetics.

The assay protocol for enzyme kinetics monitoring is shown in Figure 2, indicating the step-wise process of the reaction–mix solution’s preparation and measurement. The 1× PBS was first pipetted into a 5 mL Eppendorf tube, followed by 1 mL of L-Dopa solution, and then followed by the tyrosinase solution (depending on the volume configuration); the solution mixture was then vortexed for about 3 s. The total volume of the reaction–mix solution was 3 mL, which was subsequently pipetted into a cuvette. Respective cuvettes were then placed into a standard table-top spectrophotometer and the 3D-printed spectrophotometer simultaneously for measurement. The computer connected to these instruments logged the absorbance data from both the control instrument and the 3D-printed spectrophotometer over the specified time duration. This is repeated for all combinations of tyrosinase volume concentrations to study the reaction kinetics at different reaction rates.

### 2.5. Tyrosinase Enzyme Kinetics with Kojic Acid

Kojic acid is a common commercially used regulator to inhibit tyrosinase activity. It is also commonly used in various cosmetic products. To demonstrate the efficacy of our device to monitor tyrosinase activity inhibition using kojic acid, we modified our activity assay. Kojic acid stock solutions were prepared at different concentrations varying from 0.1 mM, 1 mM, 10 mM, 50 mM, to 100 mM. These solutions correspond to different molar concentrations of the inhibitor and were used to study the effect of inhibition on the enzyme activity over a specified time interval for a fixed tyrosinase concentration level and 50 μL inhibitor. Further, for a fixed molar concentration of 10 mM, inhibition study was performed by repeating the assay as above at different volume concentrations. For the 10 mM kojic acid, the effect of inhibition was studied at different volume concentrations for the same fixed reaction mix volume. The study was performed using 25 μL, 50 μL, 75 μL, and 100 μL of the 10 mM kojic acid solution for each of the reaction rates that were studied earlier. To keep the total volume of the reaction–mix solution constant, i.e., 3 mL, for every addition of the reagent, the volume of the PBS was reduced accordingly. All measurements were performed in triplicate.

### 2.6. Statistical Analysis

The *t*-test and Levene’s test were performed on the datasets to compare the statistical distribution between the measured values obtained using the control instrument and the 3D-printed device, including the mean and variances. The null hypotheses was: no effective difference in the mean between the datasets and equal variances between the datasets for the *t*-test and Levene’s test, respectively. We have reported the *p*-value for the measurements made considering 0.05 as the significance level, based on which the respective hypothesis was selected or rejected for each of the statistical tests.

## 3. Results

Tyrosinase enzyme activity was monitored in real time using the 3D-printed spectrophotometer and the control instrument for each of the reaction rates mentioned in the tyrosinase enzyme kinetics Section 2.4. The reaction rates were labeled as Rate1, Rate2, Rate3, Rate4, and Rate5 for the corresponding tyrosinase volumes of 60 μL, 40 μL, 20 μL, 10 μL, and 5 μL, respectively. Considering enzyme kinetics, reaction Rate1 was expected to be the fastest rate and should exhibit the highest enzyme activity. Conversely, reaction Rate5 must be the slowest rate and should exhibit the lowest enzyme activity. Enzyme activity was measured while varying both the volume concentration and molar concentration of the kojic acid, and results are presented in the following subsections.

### 3.1. Enzyme Kinetics Measurement

Enzyme kinetics were monitored based on the assay protocol discussed in Figure 2. The enzyme kinetics were monitored using both the 3D-printed spectrophotometer and the control instrument spectrophotometer. Both instruments collected absorbance measurements every 1 s. All measurements were done in triplicate.

**Figure 3:**
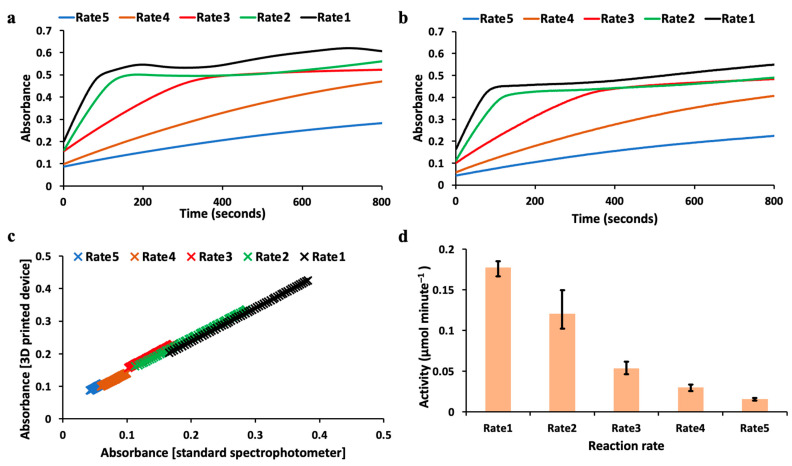
Enzyme kinetics measured at different reaction rates using the 3D-printed spectrophotometer and reference spectrophotometer are indicated. (**a**). Enzyme kinetics (average absorbance value vs. time) monitored using the reference table-top spectrophotometer. (**b**). Enzyme kinetics (average absorbance value vs. time) monitored using the 3D-printed spectrophotometer. (**c**). Correlation plots between the reference table-top spectrophotometer and the 3D printed spectrophotometer for the kinetics activity monitored at different reaction rates. Rate 1: y = 0.87 ∗ x; R^2^ = 0.9995; Rate 2: y = 0.81 ∗ x; R^2^ = 0.9981; Rate 3: y = 0.71 ∗ x; R^2^ = 0.9984; Rate 4: y = 0.66 ∗ x; R^2^ = 0.9977; Rate 5: y = 0.53 ∗ x; R^2^ = 0.9978; (**d**) Activity calculated using the 3D-printed spectrophotometer at different reaction kinetics rates with error bars. All measurements were done in triplicate.

Figure 3a shows the results of tyrosinase kinetics monitoring using the control laboratory spectrophotometer for all reaction rates mentioned above. Kinetics monitoring relied on measuring absorbance values over time. Rate5 demonstrated the lowest activity as seen by its slowly varying absorbance measurement with time, as it will take more time to hit the saturation limit. However, Rate1 reached saturation relatively sooner compared to other rates, demonstrating the highest activity, as shown in Figure 3a. This is followed by Rate2, and so on, indicating the reduction in the maximum number of available active sites for the enzyme to react and catalyze the substrate. Similarly, Figure 3b shows the results of tyrosinase kinetics monitoring using the 3D-printed spectrophotometer for all the reaction rates. Figure 3b shows the similar activity and rate of reaction trends as shown by the results presented in Figure 3a. The results shown in Figure 3a,b are the average absorbance values that were measured. Figure S1 in Supplementary Information shows the enzyme kinetics (absorbance values vs. time) measured for all repeats. The average and the variation (depicted by the band) in the measurements obtained using the reference spectrophotometer for each of the reaction rates are shown as well. Similarly, Figure S2 shows the measured enzyme kinetics (absorbance values vs. time) with variation in the measurements obtained using the 3D-printed spectrophotometer. Further, the rate of the reaction was numerically determined by considering the initial part of the absorbance curves. By correlating the kinetics measured using both the 3D-printed spectrophotometer and the control instrument, the absorbance values were plotted against each other for the region where there is a maximum rate of change of absorbance value with respect to time. Figure 3c shows the correlation plot for the kinetics of tyrosinase measured using both instruments at all reaction rates used in this study. The correlation plot shows the linear fit of the data points and presents high correlation between both instruments at all reaction rates. The correlation coefficient is measured to be 0.99 for all cases. The coefficients for the linear fit and equations are indicated in the captions of Figure 3c. The *p*-values obtained for the measured activity values at all the reaction rates, using both the instruments for Levene’s test and *t*-test, are 0.9131 and 0.9441, indicating no differences in the mean and variance between the datasets by accepting the null hypothesis.

Figure 3d shows the bar plot of the calculated enzyme activity for each reaction rate. The enzyme activity calculation was performed for the region before the absorbance saturation point was reached. Each of the reactions was repeated in triplicate, and the error bars for the enzyme activity measured are indicated in the plot shown in Figure 3d. The activity that is measured per unit minute reduces with corresponding decrease in the enzyme volume concentration. It can also be observed that the enzyme activity significantly decreases from Rate3 onwards. This indicates that the tyrosinase affinity to bind to the substrate decreases as the enzyme volume concentration is reduced. Table S1 consists of the final activity value that was measured using the 3D-printed spectrophotometer and the control instrument from the triplicate measurements performed without inhibition for all five reaction rates.

### 3.2. Enzyme Kinetics Measurement under the Influence of Kojic Acid

In a second study, each of these reactions was performed at different volume concentrations of the kojic acid to study the effect of inhibition on the enzyme activity performed. The assay protocol used for this study is similar to Figure 2, with a minor modification. Similar to the protocol shown in Figure 2, the reaction–mix sample was prepared by adding PBS, L-Dopa, and tyrosinase; however, now, the kojic acid was added towards the last step before the reaction–mix was vortexed for 3 s. Subsequently, the reaction mix was transferred into microcuvettes and then placed into both 3D-printed and laboratory instruments simultaneously for the inhibition study.

**Figure 4:**
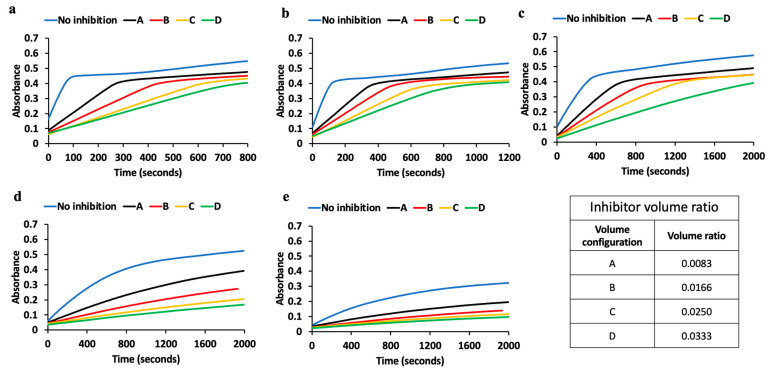
Enzyme kinetics measured using the 3D-printed spectrophotometer without inhibition and with different degrees of inhibition (controlled by the volume concentration of the inhibitor shown in the inset table). (**a**) Enzyme kinetics monitored at Rate1 being the fastest rate and at a different volume concentration of the inhibitor. (**b**) Enzyme kinetics monitored at Rate2 without inhibition and at a different volume concentration of the inhibitor. (**c**) Enzyme kinetics monitored at Rate3 without inhibition and at a different volume concentration of the inhibitor to inhibit the enzyme activity. (**d**) Enzyme kinetics monitored at Rate4 without inhibition and at a different volume concentration of the inhibitor to inhibit the enzyme activity. (**e**) Enzyme kinetics monitored at Rate5 being the slowest without inhibition and at a different volume concentration of the inhibitor to inhibit the enzyme activity. The table indicates different volume ratios of kojic acid used in the 3 mL reaction–mix solution.

The inhibition study of tyrosinase was performed using both the 3D-printed spectrophotometer and the standard control instrument. The results measured using the 3D-printed spectrophotometer are shown in Figure 4. Each of the reaction rates (Rate 1 to 5) shown in Figure 3a,b was inhibited here by using kojic acid at 10 mM concentration. Further, all reaction rates were also performed at different volume concentrations of the inhibitor. Volume configurations A, B, C, and D indicated in Figure 4 represent 25 μL, 50 μL, 75 μL, and 100 μL, respectively, of the 10 mM kojic acid inhibitor in the total 3 mL fixed volume reaction–mix solution. Figure 4a indicates the enzyme kinetics of tyrosinase without inhibition and with inhibition at different volume concentrations of the kojic acid used as inhibitor at reaction Rate1. Similarly, Figure 4b indicates the enzyme kinetics measured without inhibition and with inhibition at reaction Rate2. The enzyme kinetics of tyrosinase measured at reaction rates Rate3, Rate4, and Rate5 are shown in Figure 4c–e, respectively, without inhibition and with inhibition at different volume concentrations of the inhibitor. All these measurements were performed in triplicate. It is clearly indicated that the reaction kinetics slow down for every step of the inhibitor that was added for each reaction rate performed. Figure S3 consists of the measurements from the control laboratory instrument for the same sequence of assays performed to generate Figure 4.

**Figure 5:**
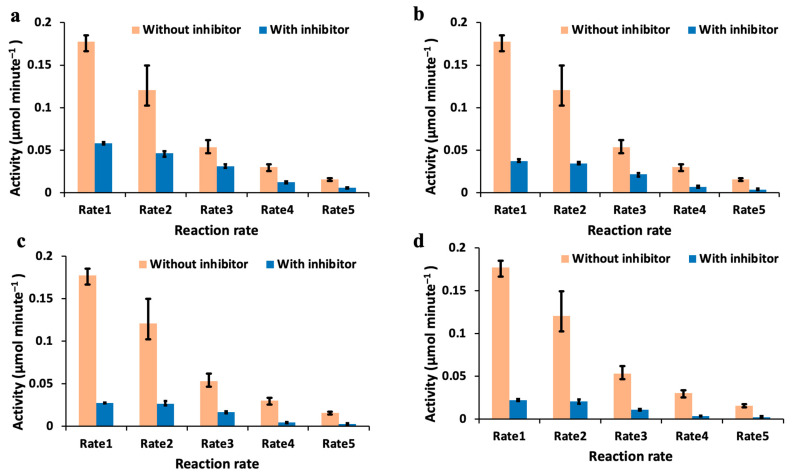
The activity calculated from the enzyme kinetics monitored over time by the 3D-printed spectrophotometer is shown here. This activity measured under the influence of different volume concentrations of the inhibitor is compared with the activity measured without any inhibitor at different reaction kinetics rates. (**a**) The activity for volume concentration A of the inhibitor is compared with the activity without any inhibition at different kinetics rates, including the error bars. (**b**) The activity for volume concentration B of the inhibitor used is compared with the activity without any inhibition at different kinetics rates. (**c**) The activity for volume concentration C of the inhibitor used is compared with the activity measured without any inhibition at different kinetics rates. (**d**) The activity for volume concentration D of the inhibitor used is compared with the activity measured without any inhibition at different kinetics rates.

In Figure 5, the effect of the inhibitor on enzyme activity measured using the 3D-printed spectrophotometer is shown. The enzyme activity was calculated from the kinetics profile shown in Figure 4. The enzyme activity measured for each volume concentration of the inhibitor was compared without inhibition for the same reaction rate in Figure 5. It was observed that for each volume concentration of the kojic acid, the enzyme activity for each reaction rate (governed by the tyrosinase volume concentration) is reduced, indicating the inhibition. Figure S4 shows the bar plots from the activity data measured using the control instrument for each of the reaction rates with and without inhibition. The *p*-values for the activity measurements at reaction Rate1 using both the instruments for different volume concentrations of the inhibitor are 0.9564 and 0.9141 for Levene’s and *t*-test, respectively. For Rate2, *p*-values were 0.9749 and 0.9262 for Levene’s and *t*-test, respectively. For Rate3, *p*-values were 0.9295 and 0.9443 for Levene’s and *t*-test, respectively. For Rate4, *p*-values were 0.9210 and 0.9452 for Levene’s and *t*-test, respectively. Similarly, for Rate5, *p*-values were 0.9575 and 0.9725 for Levene’s and *t*-test, respectively. From these values obtained, the null hypotheses are accepted, indicating that the datasets have statistically no difference in the variances and mean. The data and the calculations performed to measure enzyme activity are shown in the supplementary tables. Tables S2–S6 consists of the activity data for reaction Rate1, Rate2, Rate3, Rate4, and Rate5, respectively, and are derived from the raw data collected using both the control instrument and the 3D-printed spectrophotometer. The error bars for these measurements are calculated from the three trials of the measurements made from the same reaction rates and are also indicated in the plots.

### 3.3. Inhibition Study at Different Molar Concentrations of Kojic Acid

While the previous section discusses the effect on the enzyme activity based on the volume concentration of the kojic acid and for a fixed molar concentration, another study was performed to explore the effect of inhibition based on different molar concentrations of kojic acid. Here, kojic acid solution was prepared at different molar concentrations varying from 0.1–100 mM.

**Figure 6:**
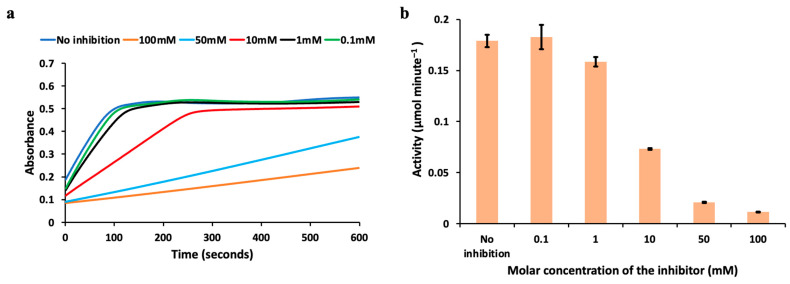
The effect of the molar concentration of the inhibitor on the enzyme kinetics at reaction Rate1 is shown in this figure. (**a**) Enzyme kinetics monitored over time at Rate1 under the influence of the inhibitor at different molar concentrations. (**b**) Activity calculated with and without an inhibitor (at different molar concentrations) for the fixed volume concentration is shown with error bars.

The enzyme kinetics monitored over time under the influence of kojic acid at different molar concentrations at reaction Rate1 and their respective enzyme activities are shown in Figure 6a,b. This study was performed for a fixed 50 μL volume concentration of the kojic acid. It was observed that the 0.1 mM concentration did not produce any statistically significant inhibition, as the reaction rate is comparable with the sample without inhibitor. The measured enzyme activity with no inhibitor is 0.1824 ${\mu \mathrm{mol}\;\min}^{-1}$, while the measured enzyme activity for the reaction with inhibitor used is 0.1826 ${\mu \mathrm{mol}\;\min}^{-1}$, which is comparable to the activity measured without an inhibitor for this reaction rate. Enzyme activity was measured to be 0.1584 ${\mu \mathrm{mol}\;\min}^{-1}$ for the reaction with 1 mM kojic acid used in the reaction–mix solution, indicating the activity to be slower than the one without an inhibitor. Similarly, enzyme activity was measured to be 0.0732 ${\mu \mathrm{mol}\;\min}^{-1}$, 0.0209 ${\mu \mathrm{mol}\;\min}^{-1}$, and 0.0115 ${\mu \mathrm{mol}\;\min}^{-1}$ for the reactions with 10 mM, 50 mM, and 100 mM kojic acid concentrations, respectively, indicating further reduction in the activity. The error bars for each of the reactions are also indicated in the Figure 6b.

### 3.4. Performance Evaluation

Finally, enzyme activity for all the reaction rates is compared between the 3D-printed spectrophotometer and the control instrument spectrophotometer. Enzyme activity is calculated for reaction Rates1 through 5 using the 3D-printed spectrophotometer and the control instrument spectrophotometer in the region where the enzyme was not saturated per unit time.

**Figure 7:**
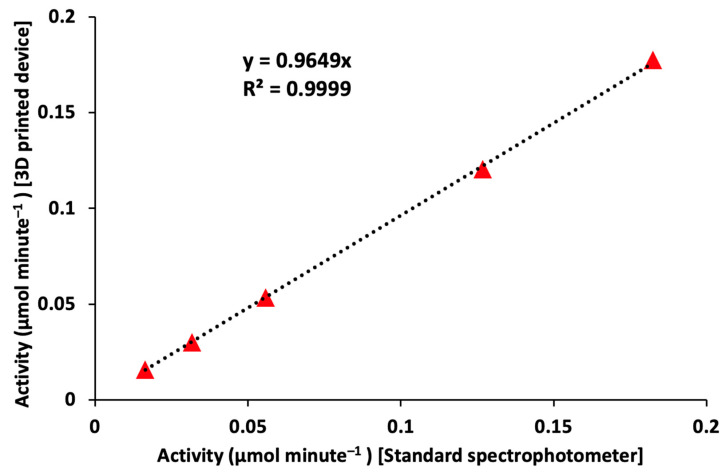
Correlation plot of the measured enzyme activity at different reaction rates between the control instrument spectrophotometer and the 3D-printed spectrophotometer.

The correlation between the enzyme activity measured using the 3D-printed spectrophotometer and the control instrument is shown in Figure 7. Enzyme activity was calculated at each of the reaction rates mentioned above without an inhibitor. In a comparative analysis between measurements obtained from 3D-printed and laboratory spectrophotometers, the correlation coefficient is obtained to be 0.9999, indicating an excellent linear fit. This proves the performance of the portable 3D-printed spectrophotometer to be comparable with the control laboratory instrument and can easily be used as an alternate for applications where these laboratory instruments are inconvenient to use.

## 4. Discussion

The importance of tyrosinase in various applications underscores the importance of monitoring its activity, which was the driving factor to develop our 3D-printed spectrophotometer. We monitored tyrosinase activity under different reaction conditions using our sensor. The different reaction rates were monitored using the same sampling rate, and it was observed that Rate1, Rate2, and Rate3 reach the saturation point in the same time window as shown in the plots in Figure 3: ~0.5–0.6 absorbance value. A similar observation was made on the enzyme kinetics measured using the 3D-printed spectrophotometer, but the saturation was observed to occur at ~0.5 absorbance value. By extrapolating the absorbance value for reaction Rates4 and 5, it can also be estimated that they will reach their saturation point at ~0.5 absorbance when measured using the 3D-printed spectrophotometer.

The absorbance values measured using both instruments were compared using the correlation plot as shown in Figure 3c. For this analysis, the data points were considered before the saturation limits. The correlation coefficient was observed to be greater than 0.99 for all the reaction rates. Similarly, the slope value was also analyzed. The slope values were found to be 0.87, 0.81, 0.71, 0.66, and 0.53 for reaction Rates1, 2, 3, 4, and 5, respectively. It can be observed that the slope value reduces as the reaction rate is reduced. This is because of the maximum absorbance value reached for each of the reaction rates before the saturation point is reached. For reaction Rate4 and Rate5, the maximum absorbance value reached is about 0.1 for the range of values considered for analysis. From the raw data collected, there is a small fixed negative offset that the 3D-printed spectrophotometer generates during the measurement. This is around a 0.05 absorbance reading, which becomes significant as the reaction rate is lowered, since it gets close to the maximum absorbance value of about 0.1 at higher reaction rates. There is a trend in the slope value measured when the reaction rate is lowered due to the decrease in the maximum absorbance value, as it approaches the fixed offset generated by the 3D-printed spectrophotometer. This offset becomes more significant at reaction Rates 4 and 5 due to the maximum absorbance value of 0.1 as per the enzyme kinetics profile from Figure 4. Each of these reactions was repeated in triplicate, and the measurements were performed accordingly for statistical analysis. Figure S1 shows the variation of the absorbance value during the 3 trials of the enzyme kinetics measurements using the standard control instrument. It can be observed that the spread in the absorbance values for reaction Rates1, 2, and 3 are much higher compared to reaction Rates4 and 5. Figure S2 shows the variation of the absorbance values of the enzyme kinetics measurements using the 3D-printed spectrophotometer for the same reaction rates. It can be noted that the spread/deviation is much less compared to the control instrument.

When the enzyme was combined with the substrate, as the reaction is being catalyzed by tyrosinase, the reaction solution is at a particular substrate concentration at a given time point. The plateau that is occurring means that the tyrosinase enzyme is being saturated by reacting with all the available L-Dopa substrate. All the data points, including both the kinetics measurements performed without any inhibitor and the kinetics measurements under the influence of the inhibitor, were processed to generate the activity values for each measurement. These activity values are presented in Figure 5 for each reaction rate without inhibition, compared with activity measured under the influence of the inhibitor for all the reaction rates. These measurements were performed using the 3D-printed spectrophotometer. Similar processing was performed on the enzyme kinetics measurement performed using the control instrument. These processed data for comparison are shown in Figure S4. The activity measured by the 3D-printed spectrophotometer is plotted against the activity measured using the control instrument in Figure 7. The correlation coefficient and the slope value have been calculated and presented. It can be observed that the correlation coefficient is 0.9999, and the slope for the linear fit is 0.9649, indicating a close performance with the standard control instrument. This proves that the device can easily be used as an alternative for the control instrument at sites where the control instrument is not accessible. To compare enzyme kinetics for each of the reactions performed above, it is necessary to calculate enzyme kinetics activity. The measurement condition for the study using the inhibitor is identical to the one during the study performed without the inhibitor, except for the reaction–mix sample that consists of the inhibitor. The 3D-printed spectrophotometer is in equal performance with the kinetics measurement with the standard control instrument with and without the inhibitor. Our device can be used as a substitute for the control instrument with advantages including portability, low power operation, and faster sampling rate. This may spearhead its applications into both commercial and other research environments. One of the main challenges with the control instrument is monitoring kinetics at a faster rate (<0.5 s, or >2 Hz). The 3D-printed spectrophotometer can be configured to perform these measurements up to 1 kHz. The device consumes 250 mW power at 5 V operation during its peak performance. This is much less than the control instrument that operates at AC 110 V. This is also much less than the power requirements of other commercially available portable spectrophotometers (e.g., 500 mW at 5 V). The 3D-printed device can be minorly configured to be used for fluorescence assay measurements. However, the 3D-printed device requires further enhancements. Currently, the 3D-printed spectrophotometer can measure at multiple wavelength channels; however, the excitation source used is a single wavelength source. In the future, a hybrid source can be developed to generate different wavelengths using the same excitation source. This can be performed using a combination of LEDs or by using a combination of light source and monochromator.

Further, currently spectrophotometers involve manual intervention to place the measurement sample inside the measurement cavity during measurement. Since the measurement is highly accurate and comparable with the control instrument, the same measurement system can be incorporated into an automated sample preparation system that can use this as a subsystem for measurements. There is also significant scope to miniaturize the optical measurement system, typically to a microfluidic level, to perform measurements of smaller volume samples and to reduce the operation power further.

## 5. Conclusions

In this study, the 3D-printed device accurately measured tyrosinase enzyme kinetics with respect to the control instrument reflected by the high correlation coefficient of 0.9999 between the activity measured using the control instrument. The measurements performed in real time on the tyrosinase enzyme using the device show its portability, low power, and accurate operation compared to the other, bulky control instruments, with a potential for future improvements. The applications of this device can also be extended to analyze other enzymes using kinetics measurements, or to monitor levels of other biomolecules, e.g., antibiotics, etc., for various applications, e.g., therapeutic drug monitoring.

## Data Availability

Relevant data is included in the main manuscript and supplementary information. Raw sensor data can be provided upon request according to institutional guidelines.

## Supplementary Materials

The following supporting information can be downloaded at: https://www.mdpi.com/article/10.3390/bios13010120/s1, Figure S1: Enzyme kinetics monitored using the standard table-top spectrophotometer at a sampling frequency of 1 Hz. (a). Rate1 measured over time and the variation in the measured value is indicated as an outline over multiple measurements. (b) Rate2 measured over time and the variation in the measured value is indicated as an outline over multiple measurements. (c) Rate3 measured over time and the variation in the measured value is indicated as an outline over multiple measurements. (d) Rate4 measured over time and the variation in the measured value is indicated as an outline over multiple measurements. (e) Rate5 measured over time and the variation in the measured value is indicated as an outline over multiple measurements; Figure S2: Enzyme kinetics monitored using the 3D printed spectrophotometer at a sampling frequency of 1 Hz. (a). Rate1 measured over time and the variation in the measured value is indicated as an outline over multiple measurements. (b) Rate2 measured over time and the variation in the measured value is indicated as an outline over multiple measurements. (c) Rate3 measured over time and the variation in the measured value is indicated as an outline over multiple measurements. (d) Rate4 measured over time and the variation in the measured value is indicated as an outline over multiple measurements. (e) Rate5 measured over time and the variation in the measured value is indicated as an outline over multiple measurements; Figure S3: The enzyme kinetics measured using the standard table-top spectrophotometer is shown without inhibition and with different degrees of inhibition (controlled by the volume concentration of the inhibitor shown in the inset table). (a) Enzyme kinetics monitored at Rate1 being the fastest rate and at different volume concentration of the inhibitor. (b) Enzyme kinetics monitored at Rate2 without inhibition and at different volume concentration of the inhibitor. (c) Enzyme kinetics monitored at Rate3 without inhibition and at different volume concentration of the inhibitor to inhibit the enzyme activity. (d) Enzyme kinetics monitored at Rate4 without inhibition and at different volume concentration of the inhibitor to inhibit the enzyme activity. (e) Enzyme kinetics monitored at Rate5 being the slowest without inhibition and at different volume concentration of the inhibitor to inhibit the enzyme activity. The table indicates different volume ratio of the kojic acid used in the 3 mL reaction mix solution; Figure S4: The activity calculated from the enzyme kinetics monitored over time by the table-top spectrophotometer is shown here. This activity measured under the influence of different volume concentration of the inhibitor is compared with the activity measured without any inhibitor at different reaction kinetics rate (a) The activity for volume concentration A of the inhibitor is compared with the activity without any inhibition at different kinetics rate including the error bars. (b) The activity for the volume concentration B of the inhibitor used is compared with the activity without any inhibition at different kinetics rate. (c) The activity for the volume concentration C of the inhibitor used is compared with the activity measured without any inhibition at different kinetics rate. (d) The activity for the volume concentration D of the inhibitor used is compared with the activity measured without any inhibition at different kinetics rate; Table S1: This table contains information regarding the different volumes of the reagents and enzymes used in the assay. Further, the enzyme activity measured using the standard table-top spectrophotometer and the 3D printed spectrophotometer at different reaction rates are shown; Table S2: The data for calculating the activity for reaction rates with and without inhibitor during multiple trials of the experiment is presented here for Rate1; Table S3: The data for calculating the activity for reaction rates with and without inhibitor during multiple trials of the experiment is presented here for Rate2; Table S4: The data for calculating the activity for reaction rates with and without inhibitor during multiple trials of the experiment is presented here for Rate3; Table S5: The data for calculating the activity for reaction rates with and without inhibitor during multiple trials of the experiment is presented here for Rate4; Table S6: The data for calculating the activity for reaction rates with and without inhibitor during multiple trials of the experiment is presented here for Rate5.

## References

[1] Wilcken B. (2001). Book review the metabolic and molecular bases of inherited disease eighth edition. edited by Charles R. Scriver, with six others. 5568 pp. in four volumes, illustrated. New York, McGraw-Hill, 2001. $550. 0-07-913035-6. N. Engl. J. Med..

[2] Lai X., Wichers H.J., Soler-Lopez M., Dijkstra B.W. (2017). Structure and function of human tyrosinase and tyrosinase-related proteins. Chem. —A Eur. J..

[3] Min K., Park G.W., Yoo Y.J., Lee J.-S. (2019). A perspective on the biotechnological applications of the versatile tyrosinase. Bioresour. Technol..

[4] Tahir K.A., Miskad U.A., Djawad K., Sartini S., Djide N.M., Indrisari M., Khaerani K., Syakri S., Masri A., Lalo A. (2021). Tyrosinase enzymes activities and sun protection factor of ethanol extract, water fraction, and n-butanol fraction of Chromolaena odorata L. leaves. Open Access Maced. J. Med. Sci..

[5] Behbahani I., Miller S.A., Okeeffe D.H. (1993). A comparison of mushroom tyrosinase dopaquinone and Dopachrome assays using diode-array spectrophotometry: Dopachrome formation vs ascorbate-linked dopaquinone reduction. Microchem. J..

[6] Fan Y.-F., Zhu S.-X., Hou F.-B., Zhao D.-F., Pan Q.-S., Xiang Y.-W., Qian X.-K., Ge G.-B., Wang P. (2021). Spectrophotometric assays for sensing tyrosinase activity and their applications. Biosensors.

[7] Braunschweig A.B., Elnathan R., Willner I. (2007). Monitoring the activity of tyrosinase on a tyramine/dopamine-functionalized surface by force microscopy. Nano Lett..

[8] Wang Z., Qiu Y., Yang T., Mao B., Huang J., Zhou S. A portable pigment concentration measurement system based on optical fiber spectrometer. Proceedings of the 2017 16th International Conference on Optical Communications and Networks (ICOCN).

[9] Kittipanyangam S., Do W., Eguchi K. Color light sensor device for light absorbance measurement device. Proceedings of the 2017 14th International Conference on Electrical Engineering/Electronics, Computer, Telecommunications and Information Technology (ECTI-CON).

[10] Murdock J., Kupcinskas R., Harjunmaa H., Kun S., Peura R.A. Minimizing errors in optical spectroscopic measurements: Utilization of Temperature Control. Proceedings of the IEEE 26th Annual Northeast Bioengineering Conference (Cat. No.00CH37114).

[11] Meyer M.T., Roy V., Bentley W.E., Ghodssi R. A microfluidic platform for optical absorbance monitoring of bacterial biofilms. Proceedings of the 2010 IEEE Sensors.

[12] Hooda P., Sami M.A., Hassan U. (2021). Point-of-care 3-D printed spectrophotometer for therapeutic drug monitoring in tuberculosis patients. IEEE Sens. Lett..

[13] Duarte L.T., Tiba J.B., Santiago M.F., Garcia T.A., Bara M.T. (2012). Production and characterization of tyrosinase activity in pycnoporus sanguineus CCT-4518 crude extract. Braz. J. Microbiol..

[14] Nguyen H.X., Nguyen N.T., Nguyen M.H., Le T.H., Van Do T.N., Hung T.M., Nguyen M.T. (2016). Tyrosinase inhibitory activity of flavonoids from Artocarpus heterophyllous. Chem. Cent. J..

[15] Wang Y., Zhang G., Yan J., Gong D. (2014). Inhibitory effect of morin on tyrosinase: Insights from spectroscopic and Molecular Docking Studies. Food Chem..

[16] Kim D., Park J., Kim J., Han C., Yoon J., Kim N., Seo J., Lee C. (2006). Flavonoids as mushroom tyrosinase inhibitors:  A fluorescence quenching study. J. Agric. Food Chem..

[17] Krishnaswamy N., Srinivas T., Rao G.M., Varma M.M. (2013). Analysis of integrated OPTOFLUIDIC Lab-on-a-chip sensor based on refractive index and absorbance sensing. IEEE Sens. J..

[18] Usuba R., Yokokawa M., Llobera A., Murata S., Ohkohchi N., Suzuki H. Integrated OPTOFLUIDIC device for the measurement of the activity of lymphocytes. Proceedings of the 2015 Transducers—2015 18th International Conference on Solid-State Sensors, Actuators and Microsystems (TRANSDUCERS).

[19] Lu D.-f., Qi Z.-m. Optical ammonia-nitrogen sensor with wide dynamic measurement range. Proceedings of the 2019 IEEE SENSORS.

[20] Pavithra N., Johri S., Ramamurthy P.C. Dopamine fluorescent sensor based on green synthesized copper oxide nanoparticles and tyrosinase. Proceedings of the 2022 IEEE International Conference on Flexible and Printable Sensors and Systems (FLEPS).

[21] Luo T., Chen Y., Wang Y. (2022). Highly sensitive and biocompatible tyrosinase sensor based on one-step synthesis of carbon dots. Mater. Today Sustain..

