# Supplementary material for: Point-of-Care Portable 3D-Printed Multispectral Sensor for Real-Time Enzyme Activity Monitoring in Healthcare Applications
Source: Biosensors (Basel). 2023 Jan 10;13(1):120. doi: 10.3390/bios13010120 (PMC9856277; doi:10.3390/bios13010120)
Supplement: Supplementary file 1 [file biosensors-13-00120-s001.zip › biosensors-2098169-supplementary.pdf]

biosensors MDPI

Supplementary Information

Point-of-Care Portable 3D-Printed Multispectral Sensor for Real-Time Enzyme Activity Monitoring in Healthcare Applications

Antony Jesuraj [1] and Umer Hassan [1,2,*]

[1] Department of Electrical and Computer Engineering, School of Engineering, Rutgers, The State University of New Jersey, New Brunswick, NJ 08854, USA
[2] Global Health Institute, Rutgers, The State University of New Jersey, New Brunswick, NJ 08901, USA
* Correspondence: umer.hassan@rutgers.edu; Tel.: +1-(848)-445-2164

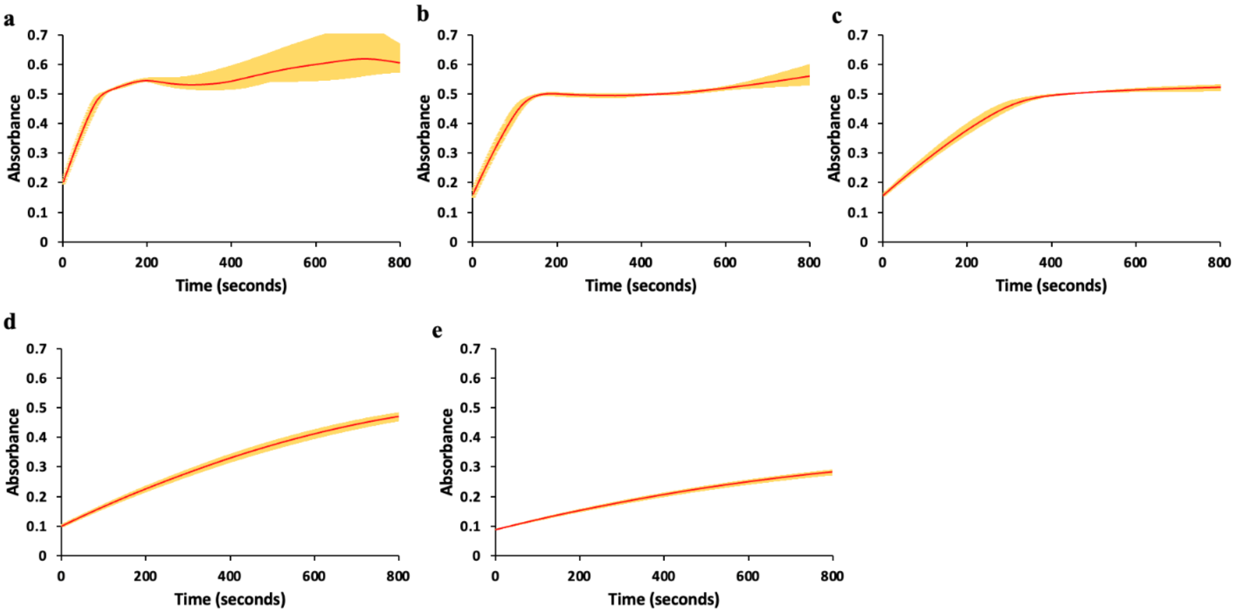

Figure S1. Enzyme kinetics monitored using the standard table-top spectrophotometer at a sampling frequency of 1Hz. (a). Rate1 measured over time and the variation in the measured value is indicated as an outline over multiple measurements. (b) Rate2 measured over time and the variation in the measured value is indicated as an outline over multiple measurements. (c) Rate3 measured over time and the variation in the measured value is indicated as an outline over multiple measurements. (d) Rate4 measured over time and the variation in the measured value is indicated as an outline over multiple measurements. (e) Rate5 measured over time and the variation in the measured value is indicated as an outline over multiple measurements.

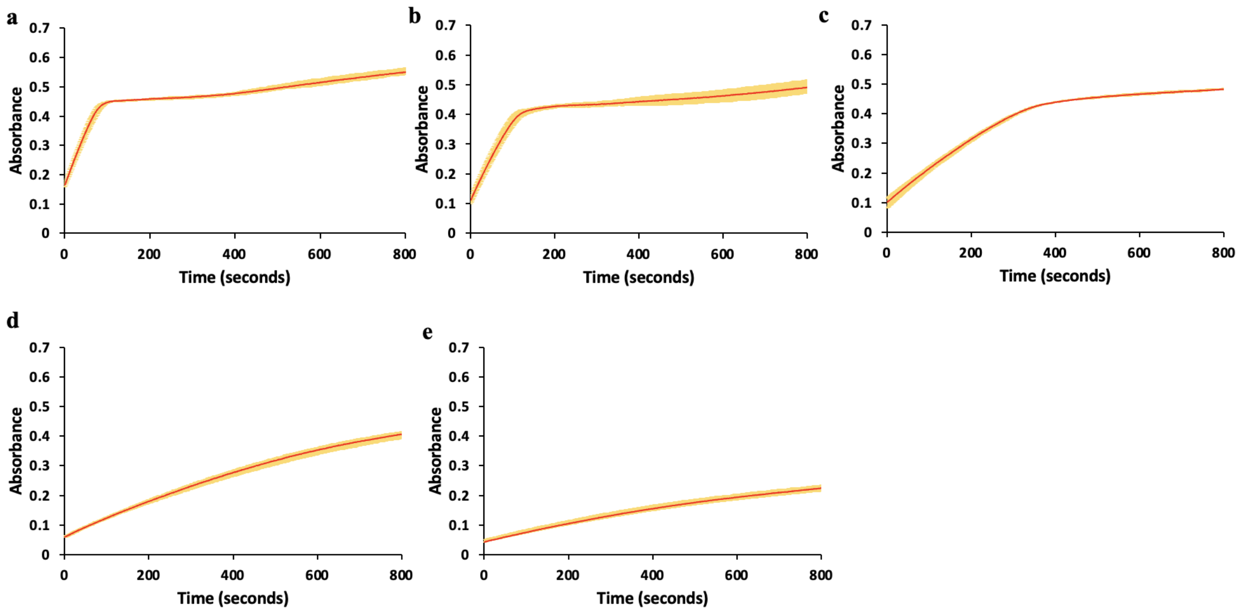

Figure S2. Enzyme kinetics monitored using the 3D printed spectrophotometer at a sampling frequency of 1Hz. (a). Rate1 measured over time and the variation in the measured value is indicated as an outline over multiple measurements. (b) Rate2 measured over time and the variation in the measured value is indicated as an outline over multiple measurements. (c) Rate3 measured over time and the variation in the measured value is indicated as an outline over multiple measurements. (d) Rate4 measured over time and the variation in the measured value is indicated as an outline over multiple measurements. (e) Rate5 measured over time and the variation in the measured value is indicated as an outline over multiple measurements.

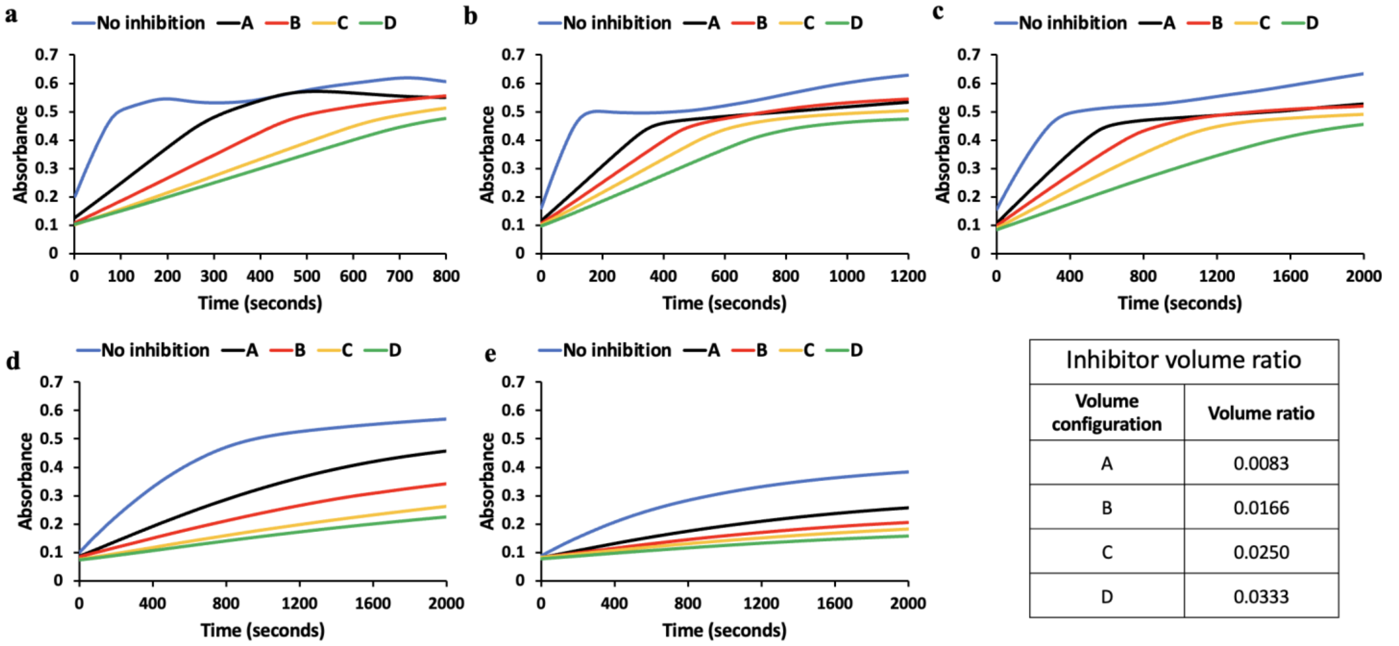

Inhibitor volume ratio	
Volume configuration	Volume ratio
A	0.0083
B	0.0166
C	0.0250
D	0.0333

Figure S3. The enzyme kinetics measured using the standard table-top spectrophotometer is shown without inhibition and with different degrees of inhibition (controlled by the volume concentration of the inhibitor shown in the inset table). (a) Enzyme kinetics monitored at Rate1 being the fastest rate and at different volume concentration of the inhibitor. (b) Enzyme kinetics monitored at Rate2 without inhibition and at different volume concentration of the inhibitor. (c) Enzyme kinetics monitored at Rate3 without inhibition and at different volume concentration of the inhibitor to inhibit the enzyme activity. (d) Enzyme kinetics monitored at Rate4 without inhibition and at different volume concentration of the inhibitor to inhibit the enzyme activity. (e) Enzyme kinetics monitored at Rate5 being the slowest without inhibition and at different volume concentration of the inhibitor to inhibit the enzyme activity. The table indicates different volume ratios of kojic acid used in the 3mL reaction-mix solution.

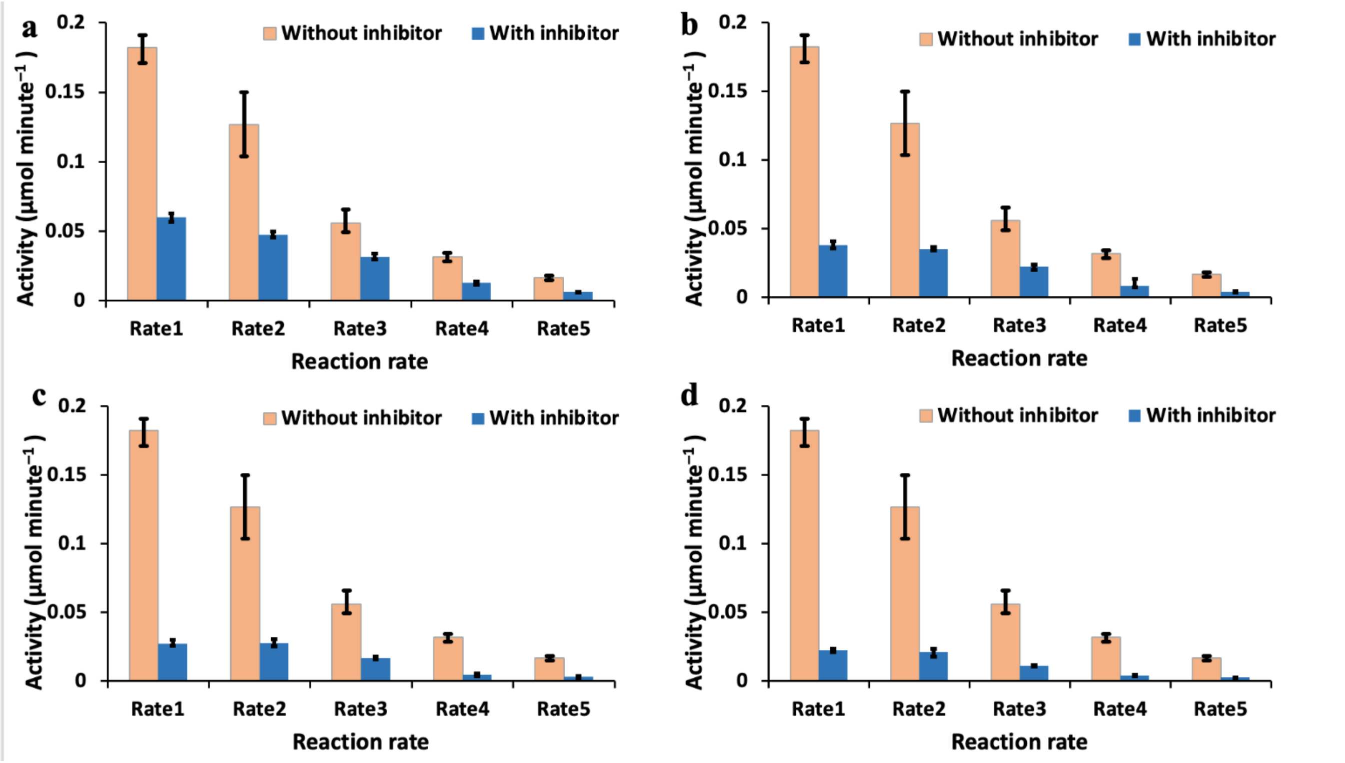

Figure S4. The activity calculated from the enzyme kinetics monitored over time by the table-top spectrophotometer is shown here. This activity measured under the influence of different volume concentration of the inhibitor is compared with the activity measured without any inhibitor at different reaction kinetics rate (a) The activity for volume concentration A of the inhibitor is compared with the activity without any inhibition at different kinetics rate including the error bars. (b) The activity for the volume concentration B of the inhibitor used is compared with the activity without any inhibition at different kinetics rate. (c) The activity for the volume concentration C of the inhibitor used is compared with the activity measured without any inhibition at different kinetics rate. (d) The activity for the volume concentration D of the inhibitor used is compared with the activity measured without any inhibition at different kinetics rate.

Table S1. This table contains information regarding the different volumes of the reagents and enzymes used in the assay. Further, the enzyme activity measured using the standard table-top spectrophotometer and the 3D printed spectrophotometer at different reaction rates are shown.

	Volume (mL)			Activity (µmol minute^{-1}) Standard table-top spectrophotometer		Activity (µmol minute^{-1}) 3D printed spectrophotometer	
Rate	PBS	LDOPA	Tyrosinase	Average	Standard Deviation	Average	Standard Deviation
1	1.940	1	0.06	0.182434	0.01019076	0.17739867	0.00986176
2	1.960	1	0.04	0.126699	0.01834497	0.1205575	0.02488914
3	1.980	1	0.02	0.055776	0.00512842	0.053369	0.00469721
4	1.990	1	0.01	0.031659	0.00186201	0.030212	0.002719438
5	1.995	1	0.005	0.016498	0.00121969	0.01577	0.00125465

Table S2. The data for calculating the activity for reaction rates with and without inhibitor during multiple trials of the experiment is presented here for Rate1.

**3D printed spectrophotometer**					
	No Inhibition	25µL kojic acid	50µL kojic acid	75µL kojic acid	100µL kojic acid
Trial 1 (0 - 60)	0.2232	0.0702	0.0473	0.0323	0.0281
Trial 1 (0 - 120)	0.0595	0.0707	0.0475	0.0319	0.0272
Trial 1 (0 - 180)	0.0031	0.0735	0.0478	0.0321	0.029
Trial 2 (0 - 60)	0.2176	0.2137	0.0439	0.0328	0.0257
Trial 2 (0 - 120)	0.0645	0.0723	0.0465	0.0325	0.0238
Trial 2 (0 - 180)	0.0031	0.0047	0.0458	0.0343	0.0274
Trial 3 (0 - 60)	0.2004	0.0717	0.0437	0.0337	0.0256
Trial 3 (0 - 120)	0.0929	0.0716	0.0476	0.0306	0.028
Trial 3 (0 - 180)	0.0078	0.0723	0.0476	0.0337	0.0283
Averaged	0.21373333	0.11853333	0.04496667	0.03293333	0.02646667
**Final Activity**	0.17739867	0.09838267	0.03732233	0.02733467	0.02196733
**Standard table-top spectrophotometer**					
	No Inhibition	25µL kojic acid	50µL kojic acid	75µL kojic acid	100µL kojic acid
Trial 1 (0 - 60)	0.2303	0.0714	0.0489	0.0311	0.0281
Trial 1 (0 - 120)	0.0768	0.0737	0.0495	0.0319	0.0299
Trial 1 (0 - 180)	0.0242	0.0747	0.0496	0.0327	0.0303
Trial 2 (0 - 60)	0.2228	0.0693	0.043	0.0328	0.0254
Trial 2 (0 - 120)	0.0903	0.0724	0.0454	0.0353	0.0269
Trial 2 (0 - 180)	0.0253	0.074	0.047	0.0359	0.0277
Trial 3 (0 - 60)	0.2063	0.0756	0.0447	0.0337	0.0276
Trial 3 (0 - 120)	0.1185	0.0775	0.0477	0.0343	0.029
Trial 3 (0 - 180)	0.0288	0.077	0.0477	0.035	0.0298
Averaged	0.2198	0.0721	0.04553333	0.03253333	0.02703333
**Final Activity**	0.182434	0.059843	0.03779267	0.02700267	0.02243767

Table S3. The data for calculating the activity for reaction rates with and without inhibitor during multiple trials of the experiment is presented here for Rate2.

**3D printed spectrophotometer**					
	No Inhibition	25μL kojic acid	50μL kojic acid	75μL kojic acid	100μL kojic acid
Trial 1 (0 - 60)	0.1683	0.0586	0.0413	0.0304	0.0253
Trial 1 (0 - 120)	0.1219	0.056	0.0417	0.0299	0.0248
Trial 1 (0 - 180)	0.0277	0.0586	0.0435	0.0313	0.027
Trial 2 (0 - 60)	0.1673	0.052	0.041	0.0324	0.0264
Trial 2 (0 - 120)	0.1246	0.0508	0.0401	0.0343	0.026
Trial 2 (0 - 180)	0.0257	0.0529	0.0418	0.0357	0.0277
Trial 3 (0 - 60)	0.1801	0.0575	0.04	0.0324	0.0214
Trial 3 (0 - 120)	0.1093	0.0569	0.0418	0.0301	0.0218
Trial 3 (0 - 180)	0.0087	0.0587	0.0418	0.0331	0.0223
Averaged	0.14525	0.05577778	0.04144444	0.03217778	0.02474444
**Final Activity**	0.1205575	0.04629556	0.03439889	0.02670756	0.02053789
**Standard table-top spectrophotometer**					
	No Inhibition	25μL kojic acid	50μL kojic acid	75μL kojic acid	100μL kojic acid
Trial 1 (0 - 60)	0.1696	0.0594	0.0425	0.0306	0.0253
Trial 1 (0 - 120)	0.1397	0.0598	0.0426	0.0323	0.0273
Trial 1 (0 - 180)	0.0363	0.0601	0.0437	0.0335	0.0277
Trial 2 (0 - 60)	0.1649	0.0524	0.0415	0.0324	0.0264
Trial 2 (0 - 120)	0.1359	0.0539	0.0438	0.0365	0.0282
Trial 2 (0 - 180)	0.04	0.0548	0.045	0.0375	0.0291
Trial 3 (0 - 60)	0.1808	0.0583	0.0411	0.0324	0.0215
Trial 3 (0 - 120)	0.125	0.0596	0.0436	0.0336	0.0234
Trial 3 (0 - 180)	0.0253	0.0601	0.0436	0.0344	0.0243
Averaged	0.15265	0.0576	0.0429	0.0337	0.0259
**Final Activity**	0.1266995	0.047808	0.035607	0.027971	0.021497

Table S4. The data for calculating the activity for reaction rates with and without inhibitor during multiple trials of the experiment is presented here for Rate3.

3D printed spectrophotometer					
	No Inhibition	25µL kojic acid	50µL kojic acid	75µL kojic acid	100µL kojic acid
Trial 1 (0 - 60)	0.0748	0.0368	0.028	0.0206	0.0134
Trial 1 (0 - 120)	0.0697	0.0367	0.0273	0.0204	0.0126
Trial 1 (0 - 180)	0.0602	0.037	0.027	0.0214	0.0143
Trial 2 (0 - 60)	0.0674	0.04	0.0226	0.0187	0.0124
Trial 2 (0 - 120)	0.0625	0.0382	0.0245	0.0182	0.0121
Trial 2 (0 - 180)	0.0598	0.0395	0.0252	0.021	0.0125
Trial 3 (0 - 60)	0.0646	0.0372	0.0272	0.02	0.013
Trial 3 (0 - 120)	0.0632	0.035	0.028	0.0183	0.0134
Trial 3 (0 - 180)	0.0562	0.0373	0.028	0.0205	0.0132
Averaged	0.0643	0.0375	0.0264	0.0199	0.013
**Final Activity**	0.053369	0.031125	0.021912	0.016517	0.01079
**Standard table-top spectrophotometer**					
	No Inhibition	25µL kojic acid	50µL kojic acid	75µL kojic acid	100µL kojic acid
Trial 1 (0 - 60)	0.079	0.0371	0.0281	0.0204	0.0134
Trial 1 (0 - 120)	0.0736	0.0375	0.0285	0.0209	0.0139
Trial 1 (0 - 180)	0.067	0.0373	0.0284	0.0214	0.0139
Trial 2 (0 - 60)	0.0693	0.0408	0.0243	0.0187	0.0128
Trial 2 (0 - 120)	0.0662	0.0401	0.0247	0.0192	0.0128
Trial 2 (0 - 180)	0.0615	0.0396	0.0251	0.0197	0.013
Trial 3 (0 - 60)	0.066	0.0375	0.0279	0.02	0.0134
Trial 3 (0 - 120)	0.0627	0.0371	0.028	0.0199	0.0138
Trial 3 (0 - 180)	0.0591	0.0366	0.028	0.0199	0.014
Averaged	0.0672	0.0382	0.027	0.02	0.0134
**Final Activity**	0.055776	0.031706	0.02241	0.0166	0.011122

Table S5. The data for calculating the activity for reaction rates with and without inhibitor during multiple trials of the experiment is presented here for Rate4.

3D printed spectrophotometer					
	No Inhibition	25µL kojic acid	50µL kojic acid	75µL kojic acid	100µL kojic acid
Trial 1 (0 - 60)	0.0381	0.0162	0.0086	0.0054	0.0048
Trial 1 (0 - 120)	0.0336	0.0162	0.0081	0.0043	0.0042
Trial 1 (0 - 180)	0.0308	0.0155	0.0096	0.0056	0.0037
Trial 2 (0 - 60)	0.0403	0.0131	0.0099	0.0043	0.0036
Trial 2 (0 - 120)	0.0376	0.0154	0.0069	0.0062	0.0048
Trial 2 (0 - 180)	0.0346	0.014	0.0097	0.0056	0.0037
Trial 3 (0 - 60)	0.0405	0.0144	0.0073	0.0049	0.0042
Trial 3 (0 - 120)	0.0379	0.0156	0.0081	0.0062	0.0048
Trial 3 (0 - 180)	0.0342	0.0148	0.0069	0.0056	0.0037
Averaged	0.0364	0.015	0.0084	0.0053	0.0042
**Final Activity**	0.030212	0.01245	0.006972	0.004399	0.003486
**Standard table-top spectrophotometer**					
	No Inhibition	25µL kojic acid	50µL kojic acid	75µL kojic acid	100µL kojic acid
Trial 1 (0 - 60)	0.0385	0.0166	0.0089	0.0053	0.0048
Trial 1 (0 - 120)	0.0364	0.0166	0.0096	0.0054	0.0044
Trial 1 (0 - 180)	0.0342	0.0164	0.01	0.0058	0.0048
Trial 2 (0 - 60)	0.0407	0.0145	0.0087	0.0043	0.004
Trial 2 (0 - 120)	0.0385	0.0151	0.0095	0.006	0.0048
Trial 2 (0 - 180)	0.0366	0.0153	0.0098	0.0062	0.0051
Trial 3 (0 - 60)	0.0413	0.0153	0.016	0.0049	0.0044
Trial 3 (0 - 120)	0.0396	0.0162	0.0099	0.0065	0.0047
Trial 3 (0 - 180)	0.0375	0.0165	0.0099	0.0066	0.0051
Averaged	0.0381	0.0158	0.0103	0.0057	0.0047
**Final Activity**	0.031659	0.0126874	0.0082709	0.0045771	0.0037741

Table S6. The data for calculating the activity for reaction rates with and without inhibitor during multiple trials of the experiment is presented here for Rate5.

**3D printed spectrophotometer**					
	No Inhibition	25µL kojic acid	50µL kojic acid	75µL kojic acid	100µL kojic acid
Trial 1 (0 - 60)	0.0208	0.0077	0.0041	0.0024	0.0023
Trial 1 (0 - 120)	0.0208	0.0073	0.0053	0.0031	0.0029
Trial 1 (0 - 180)	0.0179	0.0074	0.0042	0.0037	0.0024
Trial 2 (0 - 60)	0.0183	0.0078	0.0059	0.0042	0.0017
Trial 2 (0 - 120)	0.0185	0.0067	0.0042	0.003	0.0023
Trial 2 (0 - 180)	0.0166	0.0062	0.0042	0.0031	0.0023
Trial 3 (0 - 60)	0.0208	0.0066	0.0053	0.0041	0.0012
Trial 3 (0 - 120)	0.0192	0.0067	0.0041	0.003	0.0041
Trial 3 (0 - 180)	0.0181	0.0074	0.0036	0.003	0.0012
Averaged	0.019	0.0071	0.0045	0.0033	0.0023
**Final Activity**	0.01577	0.00589	0.003735	0.002739	0.001909
**Standard table-top spectrophotometer**					
	No Inhibition	25µL kojic acid	50µL kojic acid	75µL kojic acid	100µL kojic acid
Trial 1 (0 - 60)	0.0219	0.0075	0.0045	0.0032	0.0023
Trial 1 (0 - 120)	0.0194	0.0073	0.0049	0.0042	0.0028
Trial 1 (0 - 180)	0.021	0.0071	0.0041	0.0041	0.0027
Trial 2 (0 - 60)	0.0219	0.0078	0.0047	0.0036	0.0031
Trial 2 (0 - 120)	0.0189	0.0075	0.0052	0.0036	0.003
Trial 2 (0 - 180)	0.0201	0.0076	0.0047	0.0034	0.0029
Trial 3 (0 - 60)	0.0192	0.0077	0.0049	0.0037	0.003
Trial 3 (0 - 120)	0.0176	0.0074	0.0053	0.0036	0.0029
Trial 3 (0 - 180)	0.0189	0.0075	0.0045	0.0036	0.0029
Averaged	0.0197	0.0075	0.0048	0.0037	0.0028
**Final Activity**	0.016498	0.00623	0.003984	0.003071	0.002324
